# Characterizing Natural Frequencies of the Hybrid III and NOCSAE Headforms

**DOI:** 10.1007/s10439-024-03498-w

**Published:** 2024-04-01

**Authors:** Kristin J. Dingelstedt, Steve Rowson

**Affiliations:** Biomedical Engineering and Mechanics, Virginia Tech, Blacksburg, VA 24060 USA

**Keywords:** Experimental modal analysis, Resonance, Frequency response function, Vibrational characteristics, Short-duration head impacts

## Abstract

The vibrational characteristics of the Hybrid III and NOCSAE headforms are not well understood. It is hypothesized that they may perform differently in certain loading environments due to their structural differences; their frequency responses may differ depending on the impact characteristics. Short-duration impacts excite a wider range of headform frequencies than longer-duration (padded) impacts. While headforms generally perform similarly during padded head impacts where resonant frequencies are avoided, excitation of resonant frequencies during short-duration impacts can result in differences in kinematic measurements between headforms for the matched impacts. This study aimed to identify the natural frequencies of each headform through experimental modal analysis techniques. An impulse hammer was used to excite various locations on both the Hybrid III and NOCSAE headforms. The resulting frequency response functions were analyzed to determine the first natural frequencies. The average first natural frequency of the NOCSAE headform was 812 Hz. The Hybrid III headform did not exhibit any natural frequencies below 1000 Hz. Comparisons of our results with previous studies of the human head suggest that the NOCSAE headform’s vibrational response aligns more closely with that of the human head, as it exhibits lower natural frequencies. This insight is particularly relevant for assessing head injury risk in short-duration impact scenarios, where resonant frequencies can influence the injury outcome.

## Introduction

Anthropomorphic test device (ATD) headforms are used to study different impact scenarios. Two popular ATD headforms are the Hybrid III and the National Operating Committee on Standards for Athletic Equipment (NOCSAE), though they have structural differences [1, 2]. The Hybrid III headform was modeled after an average American man and developed for automotive safety testing. It consists of an aluminum shell with a hollow brain cavity for instrumentation and is covered with a vinyl plastisol skin [1, 3]. The NOCSAE headform was designed to evaluate protective athletic gear, modeled after an American football player. It is constructed with a polyethylene skull filled with a glycerin bladder to function as a brain, and covered with a polyurethane skin [2, 4–6]. Both headforms have been shown to have biofidelic biomechanical responses, and each has been used extensively to study sports-related head impacts, where they perform very similarly in impact scenarios typically seen in helmeted sports [1].

Headforms are used for measuring kinematic responses for a variety of loading environments. Typically, these are used for padded impact scenarios of long duration (8–15 ms), like those seen in helmeted impacts where the impact duration is extended due to the protective padding, but can also be used in short-duration loading environments (< 5 ms), typical of a bareheaded impact with a rigid object. While the Hybrid III and NOCSAE headforms have been compared in previous studies [1, 2], little research has been done to fully understand their vibrational characteristics. Gurdjian et al. [7] hypothesized that awareness of natural frequencies is useful for short-duration impacts (< 5 ms) because these impacts have a broader frequency spectrum which can cause resonance frequency excitation and skull deformation. In impact testing, it is important to consider frequency content, since the kinematic signals from these types of impact scenarios can be influenced by the vibrational responses of the headforms. The headforms might behave differently depending on the characteristics of the impact due to the structural differences affecting their frequency responses [1, 7].

**Fig. 1:**
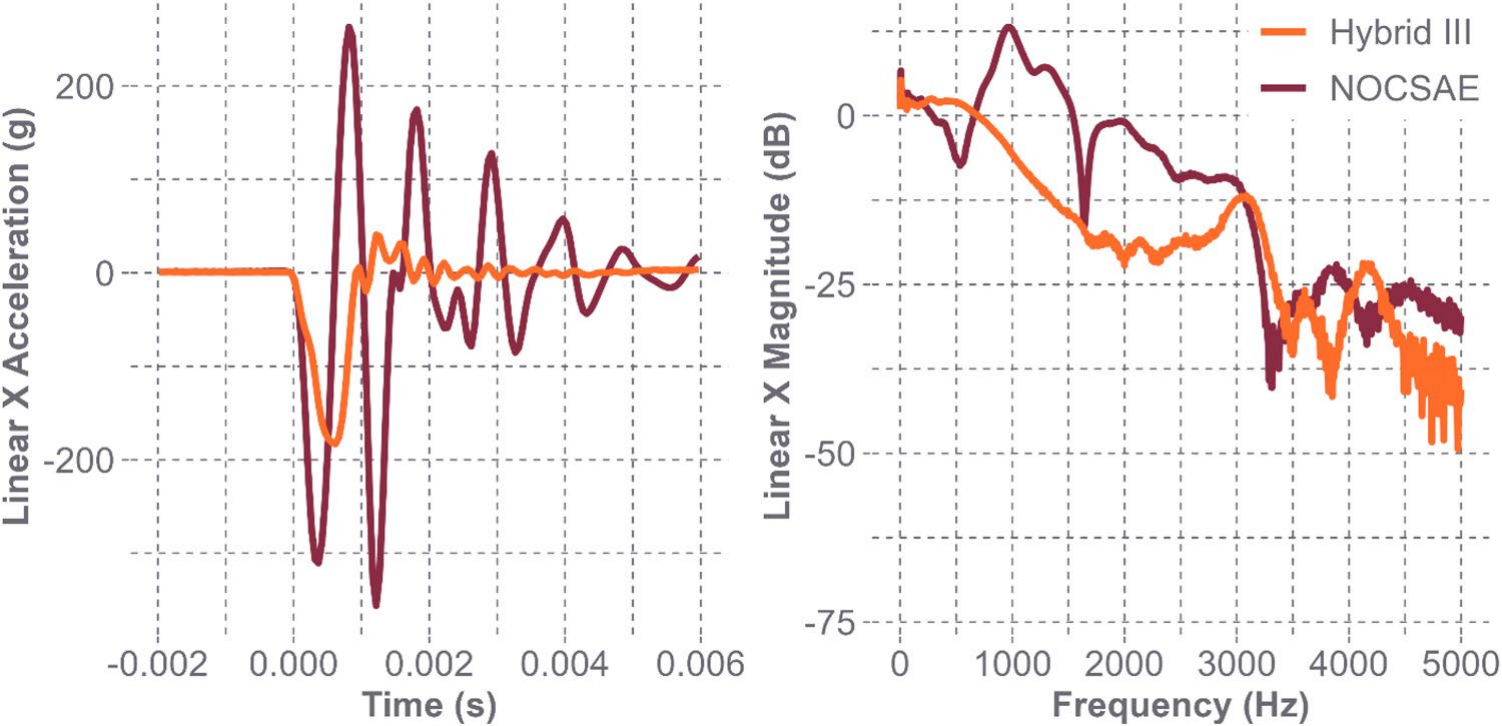
Acceleration signals comparing Hybrid III and NOCSAE headforms’ acceleration in the time (left) and frequency (right) domains for bareheaded 25 m/s baseball impacts. The NOCSAE headform’s signal contains higher acceleration magnitude and frequency content [8]

In a previous study designed to compare the responses between the Hybrid III and NOCSAE headforms for high-velocity projectile impacts, linear acceleration data were collected for baseball impacts at various speeds. Data analysis showed oscillatory responses in the NOCSAE headform, causing peak acceleration values to be more than twice that of the Hybrid III headform. A fast Fourier transform was performed, showing high-frequency peaks ranging from 500 to 1500 Hz and 1500 to 2500 Hz for 25 m/s impacts on the NOCSAE headform. Similar high-frequency responses were not seen in the Hybrid III data until approximately 3000 Hz (Fig. 1). Similar behavior was seen for 15 and 35 m/s impacts. These data suggest vibrational response differences between the headforms. While researchers have used both headforms to simulate head impacts, these data suggest that results might be more headform-dependent than previously thought for certain impact scenarios [8].

All systems have natural frequencies at which they vibrate in the absence of any driving forces. If a time-varying external load is applied to a system, and its frequency is equal to any of the system’s natural frequencies, resonance will occur. A structure is most sensitive at its natural frequencies, so as the frequency of an excitation force approaches or coincides with a natural frequency, energy is transferred very efficiently, causing the output response to be amplified. As the system oscillates at a higher amplitude, it can experience disproportionally large deflections. Modal analysis is used to study the dynamic characteristics of a system and understand its behavior during resonant vibrations. Experimental modal testing techniques involve exciting a structure with a known force and recording the response. The frequency response of the structure can be calculated and plotted, which allows the natural frequencies to be identified as the frequencies associated with the local maxima [9].

In head impacts, even relatively low-magnitude impacts can cause damage if the frequency generated by the impact corresponds to one of the head’s natural frequencies [10]. While no studies have looked at the vibrational response of ATD headforms, there have been previous attempts to characterize these dynamic properties of the human head through various experimental [7, 11–22] or computational modal analysis techniques [23–30]. The techniques used in these experimental tests were either forms of mechanical impedance tests or hammer impact tests, where the subjects were human cadaver skulls [7, 12–14, 16, 18, 20], human heads in vivo [7, 13, 15, 22], and various polymer skull models [14, 17, 21]. The objective of this study was to perform modal analysis on the Hybrid III and NOCSAE headforms to identify natural frequencies and then compare them to what has been reported for the human head.

## Methods

Modal testing methods were implemented on two headforms by using an impulse hammer to excite each headform at various locations. Data were collected from the force transducer in the impulse hammer’s tip and the accelerometers in the headforms. This allowed us to compute frequency response functions (FRFs) and determine the natural frequencies. The FRF was estimated as the ratio of the cross-power of the excitation and response signals to the auto-power of the excitation signal [31, 32]. The coherence was calculated to assess the quality of the measurements by providing insight into how much of the response was attributed to the excitation.

Impact tests were performed on a 50th percentile male Hybrid III headform and a medium NOCSAE headform. For our setup to approximately represent a free system, each headform was inverted and suspended from a metal frame with a 3/32″ diameter wire rope. This created a “free-free” boundary condition, allowing the headforms to vibrate naturally without influence from any outside structures [32]. The soft elastic system prevented any damping effects that could occur from attaching the headforms to a rigid neck. The headforms were instrumented with three linear accelerometers (Endevco 7264B-2000; Endevco Corp., San Juan Capistrano, CA) at their respective centers of gravity.

An impulse force test hammer (Model 086C04; PCB Piezotronics, Depew, NY), hereafter referred to as an impact hammer, was used to excite the headforms. The stiffness of the impact hammer’s tip dictates the input frequency spectrum. A harder tip has a shorter impulse duration, allowing for the excitation of a broader frequency range [33]. Therefore, we used a stainless-steel tip on the impact hammer. According to the specifications of the impact hammer, this hard tip is designed to excite frequencies up to 5000 Hz. However, this assumes that the structure being impacted is a harder elastic material that transmits vibrations easily, which is typical for most modal testing scenarios. The stiffness and other surface material properties of the object being impacted also affect the bandwidth [34]. The outer layers of both headforms are soft polymers, so while they behave like rigid bodies for classic impact testing, local deformations occur when hitting the headforms with the impact hammer. This elongated the impact duration, which decreased the width of the frequency range excited. We were only able to reliably capture frequencies up to 1000 Hz. This range was considered sufficient, as we do not typically see meaningful frequencies higher than this in standard helmet and automotive safety testing.

We suspected that the Hybrid III’s skin was deforming during impact which resulted in a narrow range of frequencies being excited (< 1000 Hz). To determine the Hybrid III skull’s natural frequency, we performed additional tests on the bare Hybrid III metal shell, where the skin was removed. An identical testing procedure was followed for the modified Hybrid III headform.

**Fig. 2:**
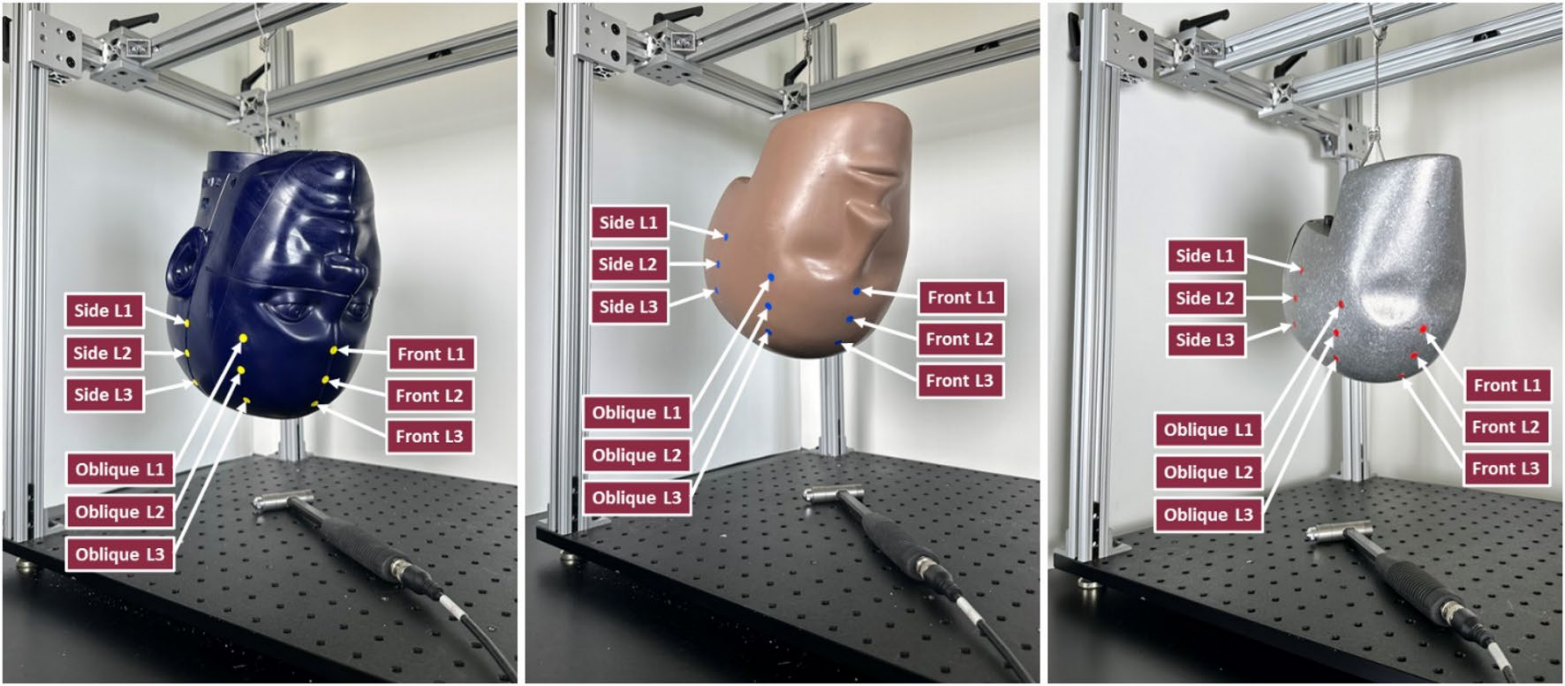
Testing setup for all impacted headforms: NOCSAE (left), Hybrid III (center), and bare Hybrid III (right) headforms. The impact hammer was used to strike each labeled location 20 times

A “roving hammer” test approach was used, where the accelerometers remained fixed and the headforms were struck at different locations (Fig. 2). We verified that each impact had a duration between 2 and 3 ms and produced similar force magnitudes (350–450 N) for the standard NOCSAE and Hybrid III headforms. Due to the hardness of the bare Hybrid III headform, once the rubber skin was removed, the impacts on this headform were much shorter (approximately 0.25–0.5 ms long) and generated forces between 650 and 850 N. A total of 540 tests were conducted, with nine locations on each headform, each struck 20 times. Multiple locations were selected to excite various possible modes in the headforms, as natural frequencies can vary with location [32, 35].

The force and acceleration signals were all sampled at 50 kHz. The resulting data were imported into MATLAB (MathWorks, Natick, MA) for analysis. The data were not filtered to avoid the possibility of missing any important frequencies. Before performing the modal analysis calculations, the data from each of the 20 trials for each location were averaged to reduce the effects of any possible random noise in each measurement [33]. We expected that the resonant frequencies would be most apparent in accelerations aligned with the resultant force vector from the hammer. However, the hammer’s resultant force vector never actually aligned with any single accelerometer’s sensing axis. To account for this misalignment, the orthogonal accelerometer signals were rotated so that a single axis aligned with the resultant acceleration vector of the impact. This allowed us to look at a single accelerometer signal for each test that was representative of the resultant acceleration vector while retaining directionality.

The computations to determine the desired metric of natural frequency were done in the frequency domain. Due to the damping effects of the headforms’ outer surfaces and the fact that the time period was sufficiently long (0.15 s; 0.05 s pre-trigger, 0.10 s post-trigger), the transient signals decayed back to zero within the sample interval. Therefore, it was not necessary to use window functions, as there was no concern about spectral leakage when the data were transformed into the frequency domain [32, 33, 36, 37]. This was true even for the bare Hybrid III headform; even though it did not demonstrate the same signal damping, it still decayed back to zero within the sampling window.

The FRFs were calculated for each location. Since the vibration response was measured as acceleration, each FRF was expressed as accelerance or inertance; this is the ratio of the acceleration spectrum to the force spectrum [32]. In these calculations, the input, *x*(*t*), was the averaged force signal or the excitation signal, and the output, *y*(*t*), was the averaged acceleration signal or the response signal. To avoid potential random errors due to noise in the recorded signals, the *H*_1_ estimator was used to determine the FRF. This estimator is applied when the output contains more noise than the input. Equation (1) displays the H_1_ FRF estimator, where *S*_*xy*_(*ω*) is the cross-spectral density between the excitation, *x*(*t*), and the response, *y*(*t*), and *S*_*xx*_(*ω*) is the auto-spectral density of the excitation [33, 38–42].

1 $${H}_{1}\left(\omega \right)=\frac{{S}_{xy}(\omega )}{{S}_{xx}(\omega )}.$$

When the accelerance FRFs were plotted, the frequencies corresponding with each local maximum were identified as the natural frequencies; this is known as the peak-picking method [43, 44]. The peaks in these plots represent areas of high amplitude magnification; the frequencies at which they occur correspond to disproportionately large oscillations in the recorded time response. This amplified response occurs because the oscillation rate of the excitation force approaches a natural frequency of the system, reaching a maximum when this rate coincides with one of the natural frequencies [31, 37, 43]. The fundamental frequency, or first mode, is the lowest frequency at which deformation occurs [7], so for the purpose of this study, we were only interested in identifying the frequency associated with the first peak of each FRF plot.

The coherence function was calculated for each location to evaluate the quality and repeatability of the measurements. The FRF calculations assume that the system is linear and time-invariant. The linearity requirement depends on the output being directly dependent on the input. An ideal excitation yields a vibrational response perfectly correlated to this force, indicated by a coherence equal to 1.0 for the sampled bandwidth. If the system is non-linear or if the signal is contaminated by excessive noise, the FRF calculations are unreliable [35, 45]. Coherence was plotted to assess the conclusions drawn from the FRF plots. The closer the coherence value was to 1.0 across the frequency range, the more confident we could be that the peaks in each FRF plot were natural frequencies and not due to measurement noise. A coherence value above 0.95 provided us with a high level of confidence that the frequencies aligned with the magnitude peaks were natural frequencies, although any coherence value greater than 0.70 is generally considered acceptable [46]. Equation (2) shows the ordinary coherence function, also known as the magnitude-squared coherence estimate, used for the coherence calculation, where *S*_*yy*_(*ω*) is the auto-spectral density of the response [47, 48].

2 $${\gamma }_{xy}^{2}(\omega )=\frac{{\left|{S}_{xy}(\omega )\right|}^{2}}{{S}_{xx}(\omega ){S}_{yy}(\omega )}.$$

## Results

### Standard NOCSAE and Hybrid III Headforms

In the Hybrid III headform, there were no natural frequencies observed below 1000 Hz. The average value for the first natural frequency of the NOCSAE headform was 812 Hz, with values ranging from 713 to 960 Hz. Table 1 displays these values by location on the headform, showing how frequency changed with position. Frontal impacts were associated with higher natural frequencies than impacts to the side or oblique regions.

**Table 1:** First natural frequency values for each impacted location on the NOCSAE headform

Impact location	Frequency (Hz)
Front L1	940
Front L2	940
Front L3	960
Oblique L1	740
Oblique L2	747
Oblique L3	747
Side L1	787
Side L2	713
Side L3	733

The subsequent figures serve as an illustrative example of our results. These plots were obtained for all nine locations on both headforms, but we picked the values from the Oblique L1 location on the Hybrid III and NOCSAE headforms, as these were the most representative of all the frontal and temporal impacts and had the clearest natural frequency peaks.

**Fig. 3:**
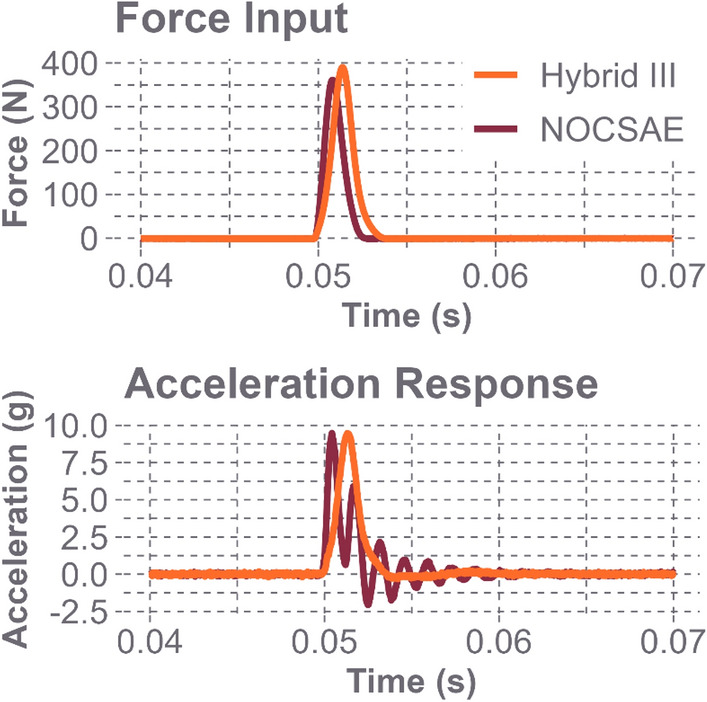
The time domain responses for both headforms at location Oblique L1. The top plot exhibits the average excitation force from the impact hammer striking the headform. The bottom plot shows the average acceleration response of the headform

The effects of the headforms’ compliant surfaces are displayed in the force and acceleration plots in Fig. 3. The Hybrid III has a softer skin than the NOCSAE headform, so it deformed more when struck at similar magnitudes with the impact hammer, making the impact duration slightly longer but resulting in a less oscillatory response. These plots support our choice to forgo the use of windows when transforming the data into the frequency domain, as the signals decay back to zero well before the end of the sample interval at 0.015 s.

**Fig. 4:**
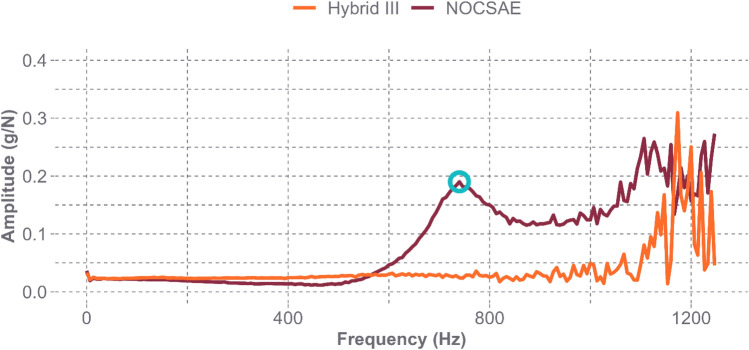
Frequency Response Functions for both headforms at the Oblique L1 location. The circled peak on the NOCSAE curve indicates the presence of a natural frequency at 740 Hz

**Fig. 5:**
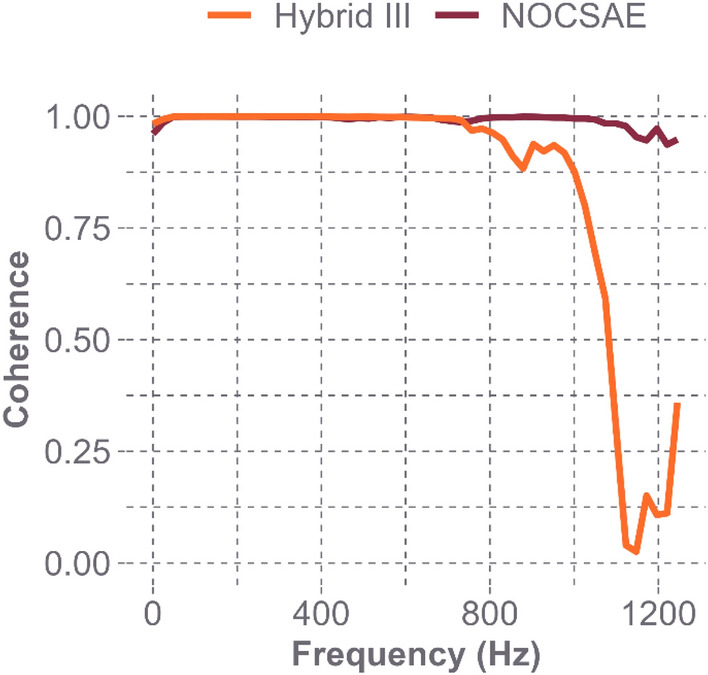
Magnitude-squared coherence estimate at Oblique L1 on both headforms. A significant drop in the coherence values occurs at frequencies greater than 1000 Hz, indicating that natural frequency identification would be unreliable beyond our bandwidth of interest

Figure 4 displays the FRF plots for both headforms at the Oblique L1 location. The FRF and coherence plots for all locations can be found in the Appendix. Peaks are seen much earlier in the frequency range for the NOCSAE headform when compared with the Hybrid III headform. We identified a natural frequency of 740 Hz for the NOCSAE headform at this location, and it is clear that there are no areas of amplified response below 1000 Hz for the Hybrid III. Noise becomes introduced at these higher frequencies, making it difficult to identify a peak as indicative of the presence of a natural frequency or simply the result of measurement noise. The assessment of the measurement quality is shown in the coherence plot in Fig. 5. The coherence values at or near 1.0 across the frequency bandwidth of interest for the NOCSAE headform indicate that the estimated modal parameters are reliable. The FRFs and coherence of the Hybrid III headform suggest that the first natural frequency of the Hybrid III headform is likely above 1000 Hz. While these plots only show the results for a single location on both headforms, the other locations had similar FRF plots, with the position of the first peak changing to correspond with the frequencies listed in Table 1. Each coherence plot provided similar confidence in the reliability of our results.

### Bare Hybrid III Headform

The tests on the bare Hybrid III headform excited a wide range of frequencies and identified natural frequencies that the rubber skin prevents from being excited. The first natural frequency of the Hybrid III’s metal skull ranged from 2459 to 3120 Hz, with an average value of 2613 Hz. Table 2 identifies these values by location on the headform.

**Table 2:** First natural frequency values for each impacted location on the bare Hybrid III headform

Impact location	Frequency (Hz)
Front L1	2980
Front L2	2520
Front L3	3120
Oblique L1	2486
Oblique L2	2473
Oblique L3	2486
Side L1	2520
Side L2	2473
Side L3	2459

For the front location, a less prominent peak was also observed around 2000 Hz (Appendix Fig. 11)

As with the above figures for the standard headform tests, the following figures serve as an example of the results for the bare Hybrid III headform by using the data from the representative Oblique L1 location. The FRFs and coherence plots for the impact tests at 9 locations of this headform can be found in the Appendix.

**Fig. 6:**
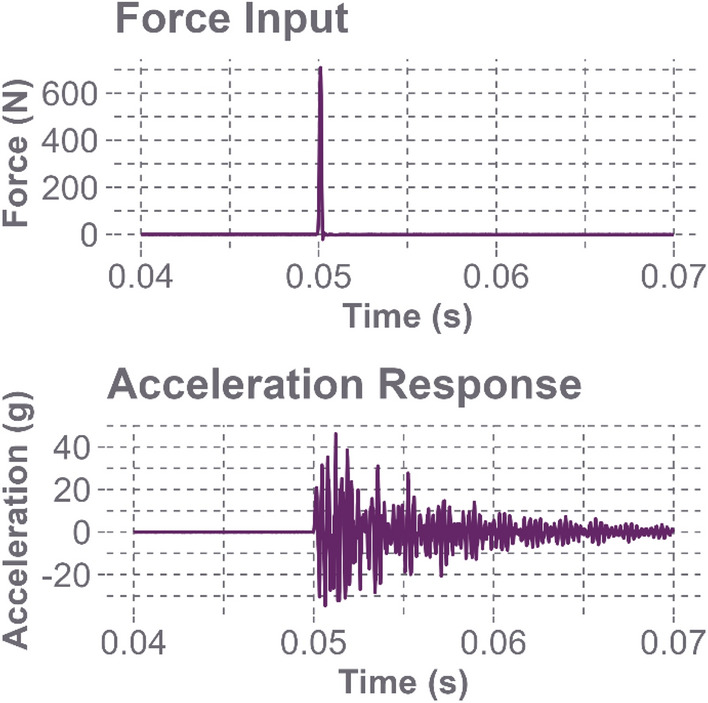
The time domain responses for the bare Hybrid III headform at location Oblique L1. The top plot shows the average excitation force from the impact hammer striking the headform’s metal surface, while the bottom plot shows the average acceleration response of the headform after impact

Figure 6 displays the response of impacting a metal headform with a metal impact hammer tip. The impact duration is much shorter, and the headform displays a greater acceleration response, both in terms of magnitude and oscillations. This signal quickly decays, demonstrating that it will return to zero by the end of the sample window.

**Fig. 7:**
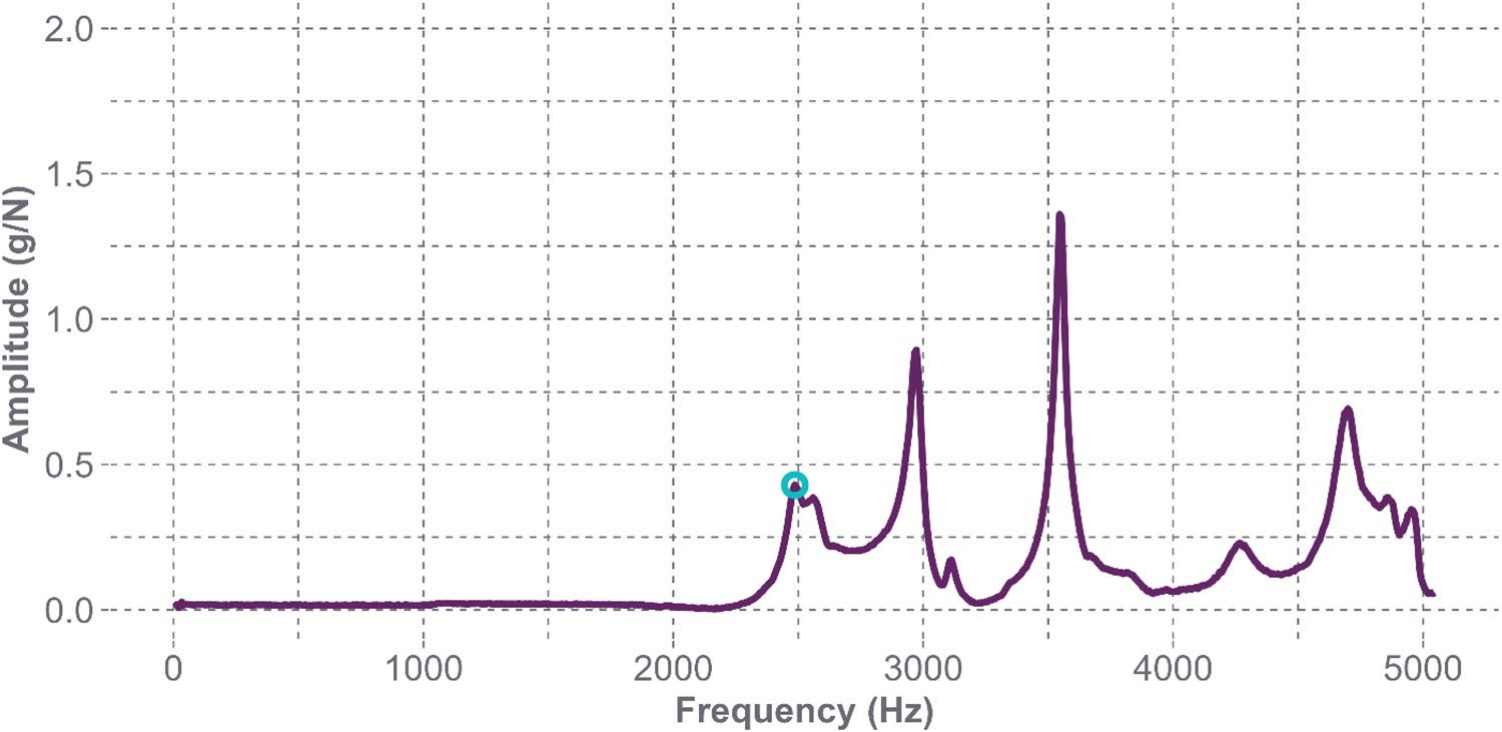
Frequency response function for the bare Hybrid III headform at the Oblique L1 location. The circled peak on the curve indicates the presence of the first natural frequency at 2486 Hz

**Fig. 8:**
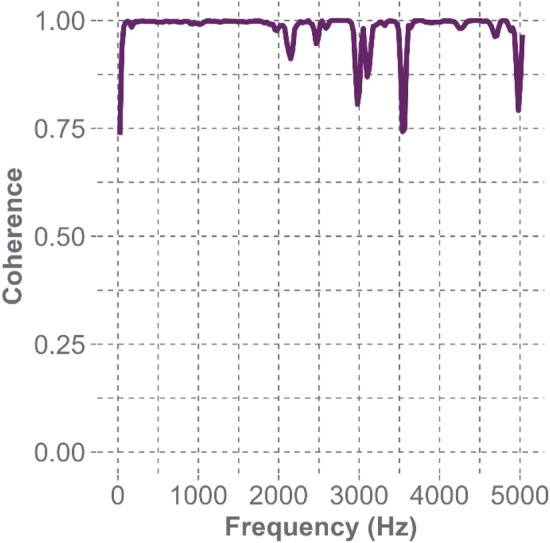
Magnitude-squared coherence estimate at the Oblique L1 location of the bare Hybrid III headform. The coherence values were approximately 1.0 up to a frequency of 2000 Hz, and remain above the acceptable value of 0.70 for the entire bandwidth of the impact hammer tip’s specified capability

The FRF plot for the bare Hybrid III headform in Fig. 7 shows very distinguishable peaks beginning around 2500 Hz. When using the impact hammer under this testing condition, measurement noise does not influence the response. Figure 8 shows a satisfactory coherence plot across the entire bandwidth of the impact hammer with the stainless-steel tip, which can capture accurate frequency responses up to 5000 Hz. The other locations had similar FRF plots, where the first peak corresponded with the frequencies in Table 2, and the coherence plots demonstrated reliability in the measurements up to much greater frequencies than seen in the standard headforms with the skins.

## Discussion

The Hybrid III and NOCSAE headforms are used to assess head injury risk in a variety of loading environments, including impacts of shorter duration. These short-duration impacts excite a larger range of frequencies than padded impacts with longer duration since time and frequency are inversely proportional. The results of this study may not have a lot of relevance for long-duration loadings, but when a wide range of frequencies are being excited with a short-duration impact, these results are important to consider. The NOCSAE headform has natural frequencies below 1000 Hz, while the Hybrid III does not.

The results from the bare Hybrid III tests support this conclusion that the first natural frequency of the Hybrid III is above 1000 Hz because when the rubber skin was removed, we were able to see natural frequencies beginning around 2500 Hz. This makes sense in the context of the baseball projectile impact results shown in Fig. 1; the Hybrid III headform did not exhibit high-frequency content until around 3000 Hz, but these were of very low magnitude and not apparent in the time domain. The rubber skin is effective at preventing resonant frequencies from being excited, which was illustrated in both the projectile tests and impact hammer tests.

To evaluate which headform’s vibrational response is more representative of the human head, we compared our results to studies done on the vibrational characteristics of humans. Both experimental and analytic tests have been performed, and although analytical results carry value, we chose to primarily focus on the results of experimental studies or analytical studies that validated their models with experimental techniques [12, 17, 20, 22]. Finite element modeling [23, 26–28] or mathematical modeling [24, 29, 30] tended to have a wider range of natural frequency values. For instance, a head–neck finite element model found the natural frequency to be 32.25 Hz [23], while a skull–brain–neck numerical model calculated the natural frequency to be 595 Hz [24]. Many experimental results have shown a natural frequency observed around 700 Hz [7, 16, 20, 22], though this varied depending on the specimen being investigated. Table 3 shows details of various experimental modal analyses performed to determine the natural frequencies of the human head. While the first natural frequencies from these tests ranged from 150 to 1385 Hz, many of the identified frequencies were similar to the values we found with our modal tests. Even though we focused more on the experimental results for our analysis, the values from modeling tests also tend to agree more with the natural frequencies of the NOCSAE headform than the Hybrid III based on our results, as they are less than 1000 Hz [23–30]. Since these previous studies have shown the human head to exhibit lower natural frequencies, the NOCSAE headform might be a more accurate representation of the human head in terms of its vibrational response. If the human head “rings” at lower frequencies that may be of interest, the NOCSAE headform should be used to better represent these impacts. This difference between headforms would only be relevant for short-duration impacts.

**Table 3:** Published natural frequency values associated with various modes in different types of skulls from experimental modal tests

Literature	Specimen	Test condition	Identified natural frequency (Hz)
Franke [13]	Dry cadaver skull and cadaver skull filled with gelatin; Living human subjects	Mechanical impedance test; electrodynamic piston applied to frontal bone	820 (cadaver) 500 (gelatin-filled) 600 (living)
Hodgson et al. [16]	Dry cadaver skull	Mechanical impedance test; frontal bone driven through impedance head by vibrating piston	360 and 950
Gurdjian et al. [7]	Cadaver skull filled with silicon gel; Living human subjects	Mechanical impedance test; sinusoidal force applied to frontal bone	313, 600, and 880 (cadaver) 300, 560, and 920 (living)
Stalnaker and Fogle [20]	Fresh, unembalmed cadaver skull	Mechanical impedance test; electromagnetic shaker attached to parietal bone	166 and 820
Khalil et al. [18]	Dry cadaver skull	Skull supported on soft rubber foam slab; excited with impulse hammer Estimated that the frequency of an in vivo head would be 53% less than the dry skull value when accounting for bone moisture, increased mass from intracranial contents, and exterior soft tissue damping	1385 651 (estimated in vivo)
Fujiwara et al. [14]	Dry cadaver skull	Skull invertedly suspended by string; excited with impulse hammer	380
Håkansson et al. [15]	Living human subjects	Mechanical impedance test; impedance head attached to skull via skin-penetrating titanium implants (used for attachment of bone-anchored hearing aids)	972
Willinger et al. [22]	Living human subjects	Frontal bone of volunteer excited with impulse hammer	150
Eslaminejad et al. [11]	Dry cadaver skull	Skull suspended by rubber bands; excited various locations with impulse hammer	496.9, 560.9, and 1246

Previous studies involving projectile impact tests performed on human cadaver skulls also support the idea that the NOCSAE headform may behave more closely to a human head in these short-duration impact scenarios [49]. Heald and Pass [50] calculated the Severity Index (SI) injury criteria for 28.6 m/s baseball impacts to the side of the head using cadaver skulls, the NOCSAE headform, and the Hybrid III headform. They found that the cadaver skull (SI = 2187) and the NOCSAE headform (SI = 2300) had more similar results when compared to the Hybrid III headform (SI = 490) [49, 50]. Additionally, another ballistic impact test conducted by Raymond et al. [51] launched a 38-mm instrumented projectile at a speed of 20 m/s to the side of several cadaver skulls. The resultant acceleration plots showed similar damped oscillations with high initial peak acceleration values to those seen in the NOCSAE signal in Fig. 1 [8]. Of the data available, comparison of cadaver and headform kinematic responses suggests that the NOCSAE headform’s vibrational response is more biofidelic in short-duration impacts.

When performing impact testing, especially with short-duration impacts, it is important to be aware of these vibrational characteristics, because when resonant frequencies occur due to the head experiencing a loading at or near one of its natural frequencies, the responses will be affected accordingly. This makes the head more vulnerable to certain impacts, regardless of their magnitudes, since a system is deformed more easily by a loading near one of its natural frequencies, increasing the potential for injury. The same impact on two distinct headforms that perform similarly in blunt impacts can be quite different in short-duration loading environments due to their unique vibrational responses. Therefore, it is important to consider this when comparing data across headforms. By understanding the intrinsic characteristics of two popular ATD headforms, informed decisions can be made in the experimental design to determine which headform will best capture the head’s frequency response relevant to the research question.

The primary limitation of this study is the relatively narrow bandwidth of frequencies that could be captured with the impact hammer on the deformable skins of the headforms. However, we believe the impact hammer is an adequate method to capture the natural frequencies of the headforms. In experimental modal testing, besides the impact hammer, the other main method of excitation is a modal shaker. The shaker transmits a driving force to the structure being tested through a thin metal rod, known as a stinger. The alignment of the shaker is important, and the geometry of the headforms could cause difficulties in attaching the stinger. Therefore, the impact hammer is the more reliable tool to avoid potential measurement inaccuracies due to misalignment issues [52]. Additionally, we are concerned with impact-driven events, so the transient excitation provided by the impact hammer is more relevant to the typical short-duration impact scenarios of interest [7, 31].

We can only obtain accurate responses up to approximately 1000 Hz due to the non-rigid headforms that deformed slightly when hit with the impact hammer. This is especially apparent in the Hybrid III headform, where the vinyl plastisol skin dampens the impact, preventing higher resonant frequencies from being excited. When the skin is removed, the bare Hybrid III skull vibrates at much higher frequencies. Thus, while we are unable to excite the natural frequencies of the Hybrid III headform as a whole, we know they will be higher than the reliable bandwidth seen in our FRF plots. The use of a modal shaker potentially could capture the natural frequencies of the Hybrid III headform, where the deformable skin would not greatly affect the excitation force, but these frequencies are expected to be similar to those found in the impact hammer test on the bare Hybrid III headform.

Since the purpose of this study is to identify the natural frequencies of these two headforms for use in the context of short-duration impacts, it is sufficient to conclude that only the NOCSAE headform exhibits natural frequencies below 1000 Hz, especially knowing that the NOCSAE headform’s natural frequencies are closer to those observed in the human head (Table 3). In short-duration impact scenarios that excite a wider range of frequencies, the NOCSAE headform is expected to behave more like the human head.

## Figures and Tables

**Fig. 9:**
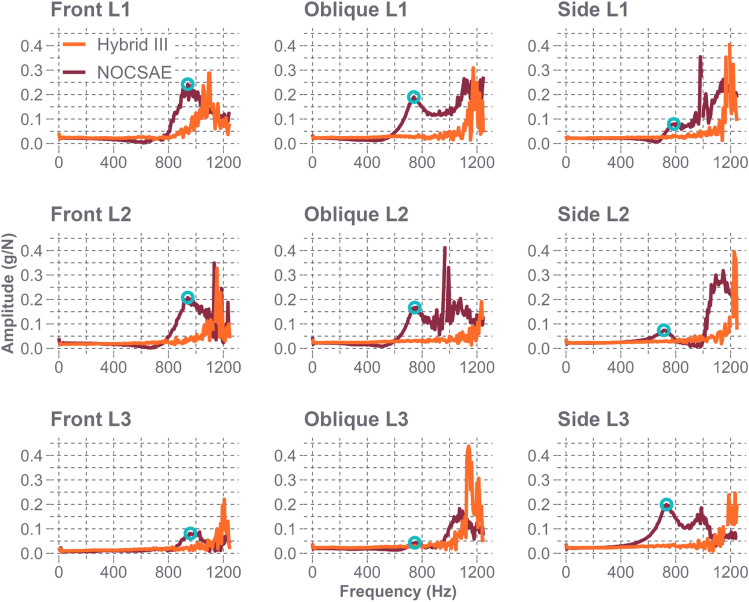
Frequency response functions for both the NOCSAE and Hybrid III headforms at each location, with the circled peaks correlating to a natural frequency

**Fig. 10:**
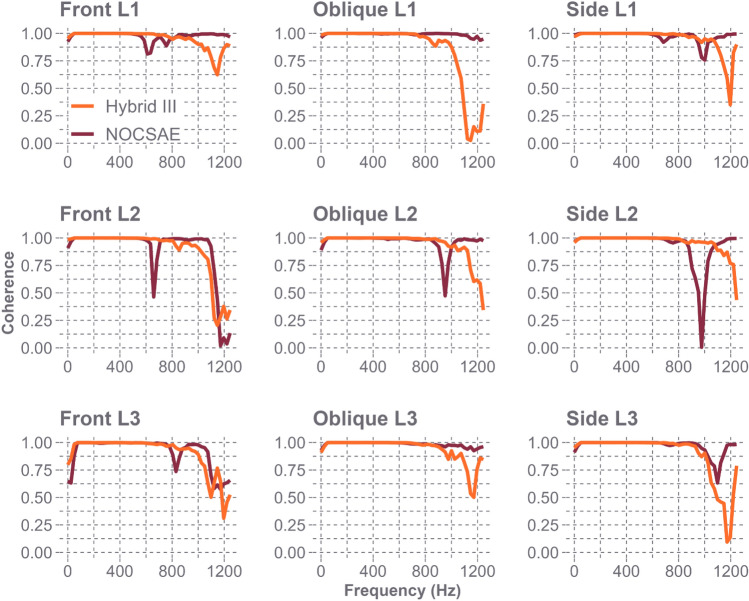
Magnitude-squared coherence estimate for both the NOCSAE and Hybrid III headforms at each location

**Fig. 11:**
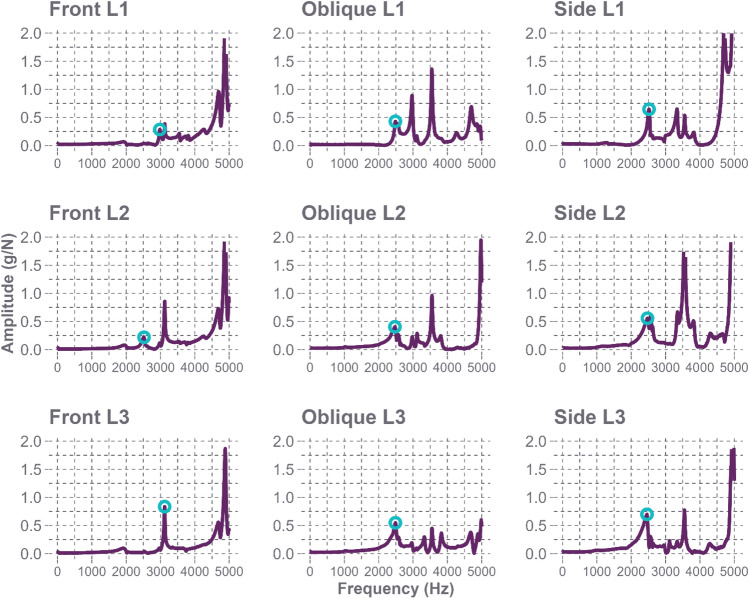
Frequency response functions for the bare Hybrid III headforms at each location, with the circled peaks correlating to a natural frequency. There was a less prominent peak around 2000 Hz seen for all of the front impact locations

**Fig. 12:**
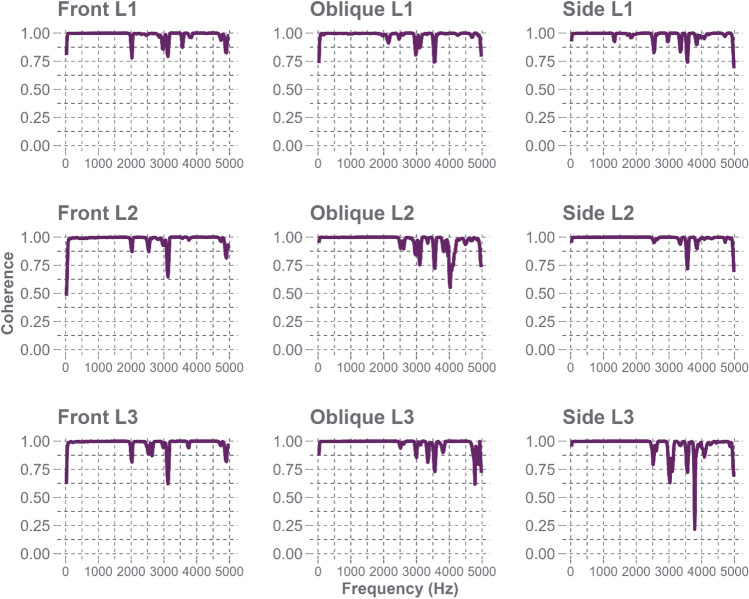
Magnitude-squared coherence estimate for the bare Hybrid III headform at each location

## Appendix

See Figs. 9, 10, 11, and 12.
